# Supplementary material for: Cortical Up states induce the selective weakening of subthreshold synaptic inputs
Source: Nat Commun. 2017 Sep 22;8:665. doi: 10.1038/s41467-017-00748-5 (PMC5610171; doi:10.1038/s41467-017-00748-5)
Supplement: Supplementary file 1 — Supplementary information [file 41467_2017_748_MOESM1_ESM.pdf]

File Name: Supplementary Information
Description: Supplementary Figures, Supplementary Methods and Supplementary References

File Name: Peer Review File

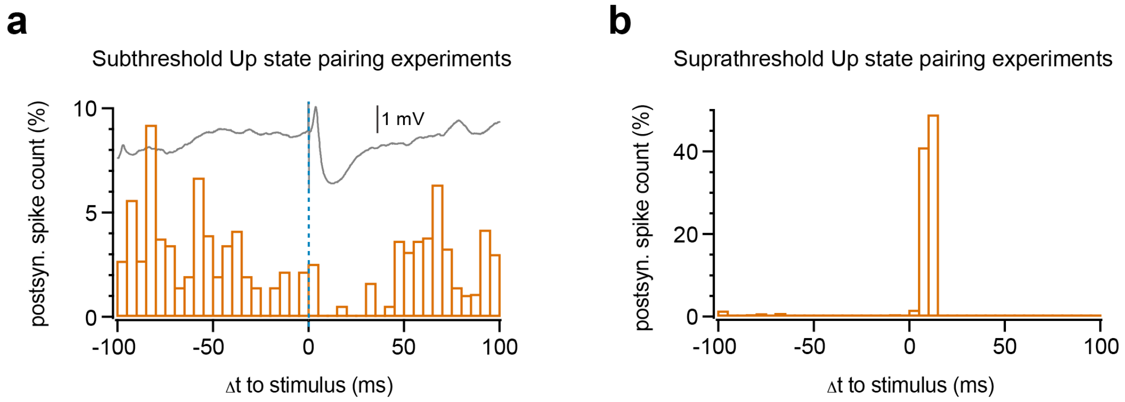

Supplementary Figure 1. Peristimulus spike-time histogram (PSTH) based on Up state pairings from Fig. 1d. (**a**) The mean PSTH was calculated for subthreshold Up state pairing experiments with Up state spike rates > 1 Hz (n=3 experiments; 50 pairings per experiment). For this, PSTHs from individual experiments were normalized to the total number of spikes in the ± 100 ms window around synaptic stimulation. The mean PSTH shows that background spiking during the Up state is largely suppressed in the +5 to +45 ms window after stimulation. The overlaid top trace is the mean Up state pairing response from a representative experiment (traces with spiking not included). It shows that extracellular stimulation during Up state periods typically induces a postsynaptic response with an inhibitory component (relative to the prestimulus Up state membrane potential). This inhibitory component is the likely cause for the coinciding suppression of spiking activity. (**b**) The equivalent mean PSTH from suprathreshold pairings (n=6) shows reliable induction of spiking within the 15 ms following synaptic stimulation. No spiking was observed during Down state pairings.

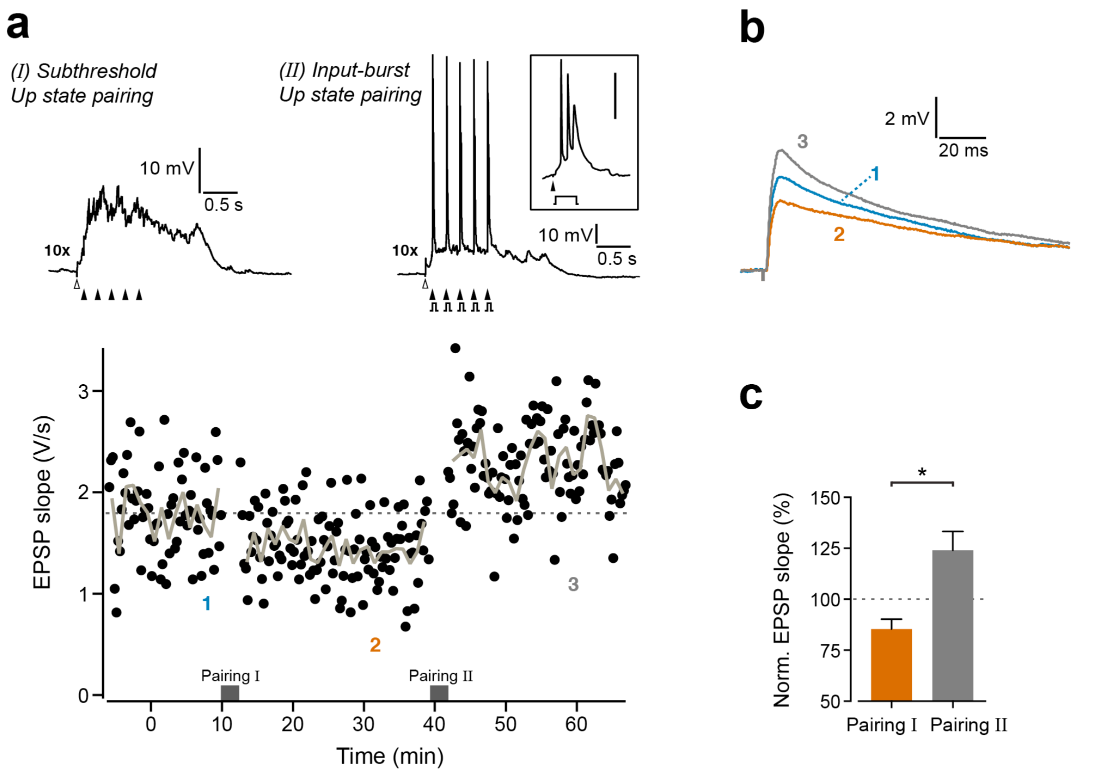

Supplementary Figure 2. Following synaptic weakening, pairing synaptic activation with spike bursts during Up states can induce synaptic de-depression. After the induction of synaptic weakening by subthreshold Up state pairings, in some experiments (two from Fig. 1 and two control recordings from Fig. 6; here pooled) a second Up state pairing protocol was applied. During the second pairing (again, stimulus trains at 5 Hz, 50 pairings in total), each extracellular stimulation was followed by a depolarizing current step (2-22 ms after the stimulus trigger) evoking 3-4 postsynaptic spikes. (**a**) Representative recording with a sample subthreshold (I) and input-burst Up state pairing (II). Arrowheads mark the timings of Up state induction (white) and the synaptic stimulation (black). The inset on the right shows one magnified input-burst pairing (scale bar: 40 mV). (**b**) EPSPs from the baseline (1), after induction of synaptic weakening (2) and after burst Up state pairing (3) from the recording shown in a (average of five traces each). (**c**) Summary of results. Pairing II successfully induced de-depression of the synapses from 85.3±4.9 % to 124.0±9.3 % of the initial baseline value (n=4, *P*<0.05; paired *t* test). Error bars show the SEM.

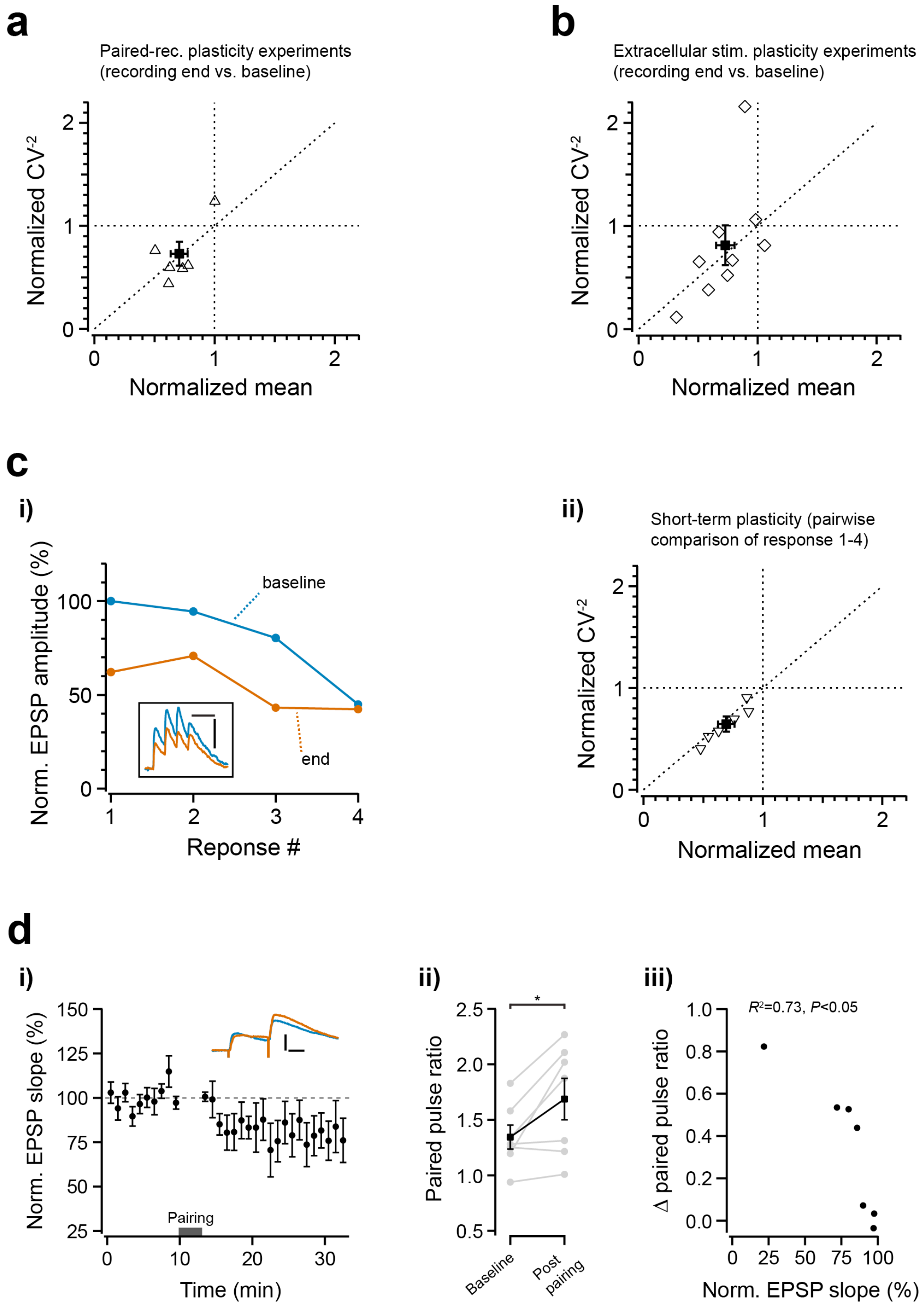

Supplementary Figure 3. Analysis of the coefficient of variation (CV) and short-term plasticity of synaptic responses is consistent with a presynaptic locus of expression. (**a**&**b**) CV analysis based on (**a**) the group of paired-recording experiments with spiking presynaptic Up sates (n=6), and (**b**) the subthreshold Up state pairing experiments shown in Fig. 1d (n=9). In both cases, most of the data points scatter around the identity line, consistent with a presynaptic component of expression. (**c**) To further support the CV analysis, in one paired-recording experiment, a train of four presynaptic spikes (inter-stimulus interval: 100 ms) was evoked throughout the recording. (i) This allowed the analysis of short-term plasticity of the evoked postsynaptic EPSPs before (inset: blue trace, baseline mean) and after induction of synaptic weakening (inset: orange trace, mean EPSPs from the period starting 25 minutes after the first Up state trigger until recording end). The response

amplitudes from the recording end were normalized to the first response of the baseline. The graph shows that, after the induction of synaptic weakening, the second response exhibits short-term facilitation as opposed to depression of the second baseline response, consistent with a decrease in neurotransmitter release probability. (Inset scale bars: 200 ms and 0.5 mV). (ii) As a control demonstrating that the CV analysis can identify a presynaptic locus of synaptic plasticity, responses 1-4 from the baseline region of the experiment from i were subjected to a CV analysis. All pairwise comparisons fell close to the identity line, which was to expect since this form of short-term plasticity has an established presynaptic origin. (**d**) To explore further the changes in short-term synaptic plasticity, additional experiments were performed using paired pulse extracellular stimulation (inter-stimulus interval: 50 ms). (i) Pooled data of normalized slope of the first EPSP (1 minute bins), showing synaptic weakening following Up state pairing (stimulus trains at 5 Hz, 50 pairings in total). Inset: representative average EPSP traces taken from the 10 minute baseline period (blue) and last 10 minutes of the recording (orange). Scale bars: 20 ms and 0.5 mV. (ii) Overall, there was a significant increase in paired pulse facilitation following Up state pairing (measured as ratio of EPSP slopes; 1.35±0.11 during baseline compared to 1.69±0.19 following pairing; n=7, $P<0.05$, paired *t* test). (iii) The change in paired pulse ratio correlated significantly with the degree of synaptic weakening (R^2=0.73, n=7, $P<0.05$; Pearson linear correlation test). Error bars show the SEM.

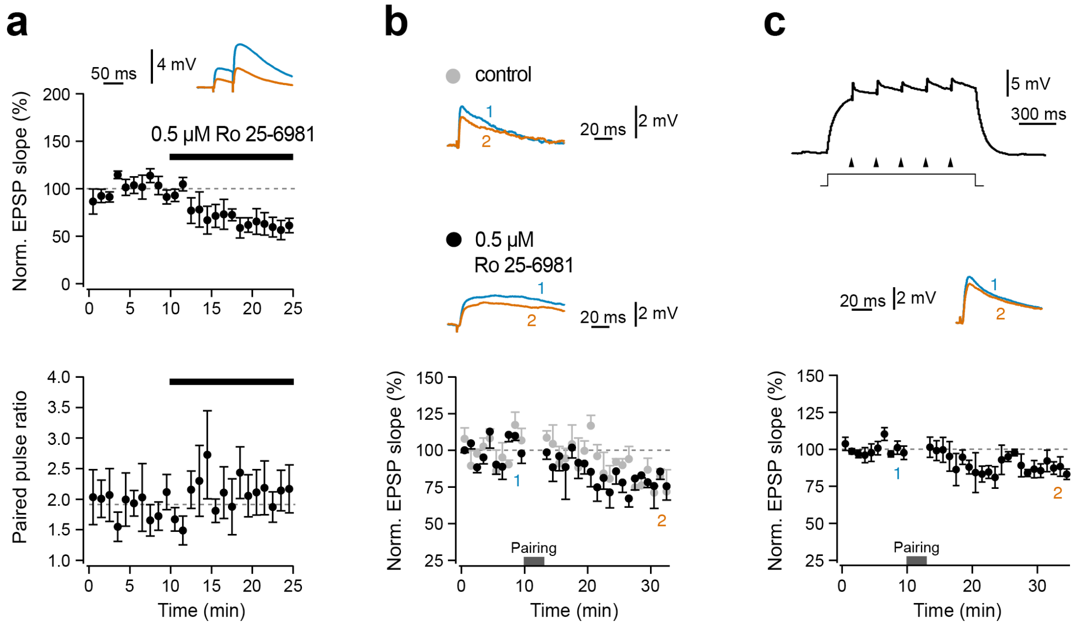

Supplementary Figure 4. Additional plasticity experiments to further elaborate the role of pre/post-synaptic NMDAR in synaptic weakening. (**a**) Presynaptic NMDAR in the mEC contain the NR2B subunit, and contribute to both the tonic and paired pulse facilitation of glutamate release[1,2]. To confirm that NR2B-containing NMDAR modulate synaptic transmission onto mEC layer III pyramidal neurons, EPSPs were recorded in response to paired pulse extracellular stimulation (inter-stimulus interval: 50 ms). Application of the NR2B subunit-selective antagonist Ro 25-6981 (0.5 μM) reduced the slope of evoked EPSPs in layer III pyramidal neurons (64.3±9.6 % of baseline; n=6, $P<0.05$, one sample *t* test; upper plot). This was not accompanied by any significant changes in the paired pulse ratio of the EPSP slopes (n=6, $P=0.45$, paired *t* test; lower plot), which could reflect a combined effect of inhibiting tonic and spillover activation of presynaptic NMDAR. (**b**) Up state pairing in the presence of 0.5 μM Ro 25-6981 induced synaptic weakening to 78.2±4.9 % (n=4), which was not significantly different from that obtained during interleaved vehicle controls (81.8±4.7 %; n=4; $P=0.69$; unpaired *t* test). Top & middle: Representative EPSPs from the baseline (1) and after pairing (2) (average of five traces each), from the vehicle control group (top) and Ro 25-6981 group (middle). Bottom: Pooled data for normalized EPSP slope (1 minute bins). (**c**) If postsynaptic NMDAR-mediated currents are important for driving synaptic weakening during Up state pairing, then this plasticity would be expected to be sensitive to postsynaptic membrane potential. Top: Stimulus trains (5 Hz, 50 in total) were paired with a depolarizing current step to mimic the typical Up state-associated somatic depolarization (mean prestimulus step amplitude: 10.6±0.84 mV; approximating the prestimulus Up state amplitude of 9.2 mV from Fig. 1d panel ii). Middle: Representative average EPSPs from the baseline (1) and after pairing (2) (average of five traces each). Bottom: Pairing stimulus trains with somatic depolarization was sufficient to induce a significant reduction of the EPSP slope to 88.9±3.6 % (n=5, $P<0.05$; one sample *t* test).

Supplementary methods

Pharmacology

For *Supplementary Fig. 4*: Ro 25-6981 was purchased from Abcam. For Up state-induced plasticity experiments in the presence of Ro 25-6981, standard aCSF was replaced with aCSF + 0.5 μM Ro 25-6981 > 30 minutes before experiment onset.

Data analysis

For *Supplementary Fig. 2*: The degree of synaptic plasticity induced by pairing I was quantified as the baseline-normalized mean response slope from the 10-minute period starting 10 minutes after pairing I. This matches the quantification in the results section. Plasticity induced by pairing II plateaued quickly; here, normalized slopes from the 15-minute period starting 5 minutes after pairing II were averaged. For *Supplementary Fig. 3*: In the CV analysis based on response short-term plasticity, the response amplitudes were approximated by the difference of the peak and the pre-response membrane potential. The response amplitudes 2-4 from the average traces were extracted by fitting an exponential curve to the decay phase of the respective preceding response. CV analyses were noise corrected.

Supplemental references

1. Berretta, N. & Jones, R. S. Tonic facilitation of glutamate release by presynaptic N-methyl-D-aspartate autoreceptors in the entorhinal cortex. *Neuroscience* **75,** 339–344 (1996).
2. Woodhall, G., Evans, D. I., Cunningham, M. O. & Jones, R. S. NR2B-containing NMDA autoreceptors at synapses on entorhinal cortical neurons. *J. Neurophysiol.* **86,** 1644–1651 (2001).
